# Remote Sensing X-Band SAR Data for Land Subsidence and Pavement Monitoring

**DOI:** 10.3390/s20174751

**Published:** 2020-08-22

**Authors:** Sadra Karimzadeh, Masashi Matsuoka

**Affiliations:** 1 Department of Remote Sensing and GIS, University of Tabriz, Tabriz 5166616471, Iran; 2 Institute of Environment, University of Tabriz, Tabriz 5166616471, Iran; 3 Department of Architecture and Building Engineering, Tokyo Institute of Technology, Yokohama 226-8502, Japan; matsuoka.m.ab@m.titech.ac.jp

**Keywords:** pavement, land subsidence, synthetic aperture radar, SAR interferometry

## Abstract

In this study, we monitor pavement and land subsidence in Tabriz city in NW Iran using X-band synthetic aperture radar (SAR) sensor of Cosmo-SkyMed (CSK) satellites (2017–2018). Fifteen CSK images with a revisit interval of ~30 days have been used. Because of traffic jams, usually cars on streets do not allow pure backscattering measurements of pavements. Thus, the major paved areas (e.g., streets, etc.) of the city are extracted from a minimum-based stacking model of high resolution (HR) SAR images. The technique can be used profitably to reduce the negative impacts of the presence of traffic jams and estimate the possible quality of pavement in the HR SAR images in which the results can be compared by in-situ road roughness measurements. In addition, a time series small baseline subset (SBAS) interferometric SAR (InSAR) analysis is applied for the acquired HR CSK images. The SBAS InSAR results show land subsidence in some parts of the city. The mean rate of line-of-sight (LOS) subsidence is 20 mm/year in district two of the city, which was confirmed by field surveying and mean vertical velocity of Sentinel-1 dataset. The SBAS InSAR results also show that 1.4 km^2^ of buildings and 65 km of pavement are at an immediate risk of land subsidence.

## 1. Introduction

Land subsidence is a gradual downward movement of the ground due, e.g., to the withdrawal of a large amount of water from underground layers [1,2,3] or mining activities [4]. When the underground space becomes empty, the surficial ground falls in on itself. The trend for ground subsidence is not usually sudden; it takes several years to be visible. The land subsidence starts slowly and spreads to adjacent areas, where it could affect agricultural, industrial, and urban activities. Land subsidence has been recognized as a serious environmental problem [5,6,7]. There are some strategies to monitor or control land subsidence, but if it progresses without the required supervision, the land could lose its functionality in the future. For example, sinkholes appear in agricultural land when the underground space becomes too large and causes a sudden collapse. Reviving such land for agricultural activities would be difficult after the failure point. Land subsidence takes place not only in agricultural land but also in urban areas. In urban areas, the land subsidence phenomenon is as complex as in nonurban areas—a mixture of geotechnical, hydrogeological, and engineering aspects [8]. Land subsidence in urban areas could damage building foundations, collapse walls, and cause damage to pavement and gas and water pipelines [9,10,11]. In recent decades, land subsidence monitoring has increased in terms of both spatial and temporal distribution. One of the conventional methods of land subsidence monitoring is precise leveling. Another conventional method is the station-based global positioning system (GPS) approach, in which each station can provide three-dimensional displacements with an acceptable accuracy [12,13]. Although the GPS method can provide higher accuracy of measurements, there is sparse coverage and the high cost of establishing stations is always debatable [14]. Remote sensing technology provides more efficient and cheaper tools such as unmanned aerial vehicles (UAV), airborne laser scanning, or airborne imagery [15,16]. These tools are efficient, but making regular measurements over a certain area is still bothersome. Alternatively, satellite remote sensing, especially synthetic aperture radar (SAR) remote sensing, which has developed in the last two decades, gathers information from the Earth regularly. For example every six days for the Sentinel-1 constellation (S1A and B), or every four days for the Cosmo-SkyMed constellation if all four of the constellation’s satellites are operational. SAR remote sensing techniques such as differential SAR interferometry (DInSAR) or small baseline subset (SBAS) can be effective for the analysis of land subsidence over large areas. In urban areas, SAR remote sensing techniques are popular and the number of studies that are trying to find correlations between engineering plans and remote sensing techniques has increased. Recent SAR remote sensing studies show that the monitoring of major urban elements such as railroads, bridges, and buildings is possible [17,18,19]. Two components of SAR images are the amplitude and the phase. Both components are used successfully to measure deformations such as earthquake damage, volcanic activity, land subsidence, and landslides [20,21,22,23,24,25,26,27,28,29,30,31,32,33,34]. However, for long-term deformations (e.g., land subsidence), interferometric methods (i.e., phase-based) are favorable. Here amplitude and phase information will be used profitably for pavement and land subsidence, respectively. Amplitude information will be used to extract buildings and roads, which stand out against a low backscattering coefficient in urban areas. We introduce a fresh idea to deal with traffic jams in urban areas using a stack of amplitude images. The phase information will also be used in SAR interferometry (InSAR) to extract vertical land displacement. If ground truth data are not available, high resolution (HR) SAR images over urban areas are necessary to extract displacement and urban elements (e.g., buildings and roads). The SAR images contain useful information for several purposes. In the next section, we present the land subsidence problem in Iran, a semi-arid to arid country, explain the geological characteristics of the study area, and discuss the HR SAR images acquired from the Cosmo-SkyMed (CSK) constellation.

## 2. Study Area and Dataset

### 2.1. Study Area

Iran is a semi-arid to arid country with two major deserts, the Kavir and Lout. According to previous studies, precipitation in the country is lower than average. Thus, the land subsidence problem in Iran’s strategic plains (e.g., Varamin, Mashahd, Tabriz, etc.) is related to the overexploitation of underground water [30,31,32,35]. Due to industrialization and the high demand for water by different sectors, water shortage in major basins of the country is becoming a major challenge for the government. Only in the capital, Tehran, and its surrounding land, has the number of water wells increased roughly eightfold between 1986 and 2012. These are legally excavated wells, but considering other illegal wells, the number of dams constructed, and other effective parameters of urbanizations. The roadmap of development of the country was built on rapid industrial and agricultural production, with no plan B for the possible water-shortage era [31,36].

**Figure 1:**
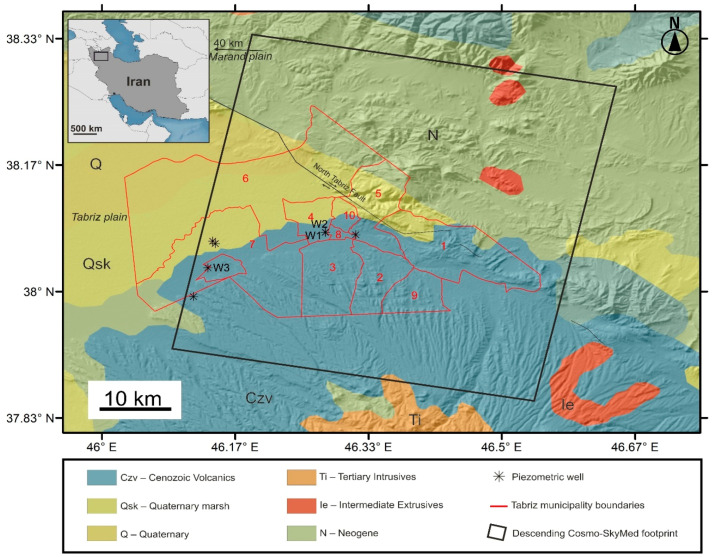
The study area with geological classes (provided by Geological Survey of Iran) on shaded relief global 30 arc second elevation data (GTOPO30). The red shapes show the 10 municipal districts of Tabriz; the black polygon is the synthetic aperture radar (SAR) footprint from the Cosmo-SkyMed (CSK) mission. Black asterisks and black lines are the piezometric sites and faults, respectively. Piezometric measurements of the W1, W2 and W3 sites are available. Must be noted that the W1 and W2 are too close to each other.

Tabriz is an industrial Iranian city located in northwestern Iran. It is the sixth most populous city of Iran, with a high demand for water for agricultural, residential, and industrial purposes. Figure 1 shows the location of the study area and its geological descriptions. The city currently has 10 municipal districts (the red shapes in Figure 1) with urban and nonurban areas. All of the municipal districts belong to four geological classes. Districts 2, 3, 8 and 9 belong completely to the Cenozoic volcanic class, while districts 1, 5, 7 belong to Cenozoic volcanic, Quaternary marsh and Neogene classes. Districts 4 and 10 are part of both the Cenozoic volcanic and Quaternary marsh classes. District 6 is situated in the Quaternary and Quaternary marsh, Cenozoic volcanic and Neogene classes. The total area of Tabriz municipality is approximately 782 km^2^, of which the largest (district 6) and smallest (district 8) districts are approximately 280 km^2^ and 4 km^2^, respectively. The largest and smallest geological classes inside the municipal boundaries are the Cenozoic volcanic (~363 km^2^), and Neogene (~61 km^2^) classes, respectively. The North Tabriz fault, a major tectonic feature (~150 km) in NW Iran, stretches in the NW-SE direction, passing through districts 1, 5, and 6. Instrumental records near the fault suggest that the fault is seismically active and mature with right-lateral strike-slip motions [37]. The North Tabriz fault’s slip rate is estimated from trenching, GPS, and InSAR methods, suggesting that the slip rate is ranging between 2 and 10 mm/year [35,38,39].

There are several studies related to InSAR land subsidence in Iran [30,31,32,35], but few of them have focused on urban areas in NW Iran [30,40]. An InSAR SBAS time series analysis of 17 Envisat ASAR images revealed that, between 2003 and 2010, rapid subsidence with a maximum rate of 20 mm/year occurred near the Tabriz thermal power plant in the Tabriz plain [30]. In the Tabriz plain, the progressive land subsidence could be harmful for structures and infrastructure such as roads. The black asterisks in Figure 1 show the location of several piezometric sites where the Regional Water Organization (RWO) regularly controls water level fluctuations in the Tabriz plain and surrounding areas. Piezometric measurements are an integrated measurement of the water level over time. The measurements could be positive or negative, depending on the reference benchmark’s water level situation. If the measurements are positive, it means that they are below the reference level (in most cases) and negative if the opposite situation occurs (artesian wells). There are hundreds of piezometric stations in the Tabriz plain, but here we only show those stations located inside the city boundaries. Using the same InSAR method, the maximum rate of subsidence for the Envisat ASAR dataset (between 2003 and 2006) and Sentinel-1 (between May and October 2015) dataset was 24 cm/year and 39 cm/year, respectively, in the Marand plain, 40 km from Tabriz [40]. Despite the successful application of the Envisat and Sentinel-1 datasets for land subsidence monitoring of the large-scale subsidence in major basins, HR SAR data on the urban scale have not been yet applied to the region. The main objective of this study is to examine the potential of HR SAR data in urban areas, not only for deformation monitoring, but also for gathering auxiliary information on the buildings and pavement by HR SAR data. Accordingly, in the next subsection and section, the CSK data description, traffic noise, pavement quality, SBAS method and extraction of vertical motion will be explained.

### 2.2. Dataset Description

For this study, SAR images were obtained from the CSK mission. The CSK constellation consists of four satellites, designed by the Italian space agency (ASI). This was a collaborative project between the Italian Ministry of Research (MUR) and the Italian Ministry of Defense. The satellites of the system are midsize sun-synchronous satellites with multimode high-resolution SAR sensors. All the CSK satellites use X-band, which is a segment of the microwave radio region of the electromagnetic spectrum (wavelength: ~3.1 cm). The revisit interval of the mission is considerably shorter than for former missions in Europe due to its constellation nature. The revisit interval of each CSK satellite is 16 days. If all the CSK satellites are in action, the revisit interval could be reduced to four days. The satellites were launched between 2007 and 2010. For the first 10 years of operation, more than 1 million SAR images were collected all over the world [41,42]. Although the perspective and the revisit interval of the constellation are ideal for monitoring natural hazards such as earthquakes, fires, and land subsidence, the spatial coverage is highly concentrated on the European territory. Some characteristics the SAR images of the CSK constellation are as follows: 1—very high-resolution (VHR) spotlight images (<3 m) for special use on monitoring of small features or individual objects (e.g., structures), 2—high-resolution Stripmap images in HIAMGE and PINGPONG modes (3–10 m) for midsize objects and routine purposes such as interferometric analysis, 3—medium- and low-resolution images (30–100 m), focusing on large-scale features [41,42]. Here, we acquired 15 HR CSK images (eight images from satellite 1, three images from satellite 2, and four images from satellite 4) in Stripmap HIMAGE mode, all from descending orbits. We had only quota for 15 HR CSK images according to the agreement between the ASI and the European Space Agency (ESA) which is a small dataset for SBAS analysis, but according Crosetto et al., typically 15–20 images are enough for time series analysis; it is even possible to use short dataset for X-band data because of higher resolution and shorter wavelengths [43]. Since the study area is arid and semi-arid, the temporal decorrelation is not a major issue in this case. The footprint dimensions of HR CSK images, as shown in Figure 1, are 40 km × 45 km, all in SCS (standard single-look complex slant) format. The SCS format’s specialties and its zero-Doppler projects make the format suitable for urban-scale applications such as change detection and deformation monitoring. The standard single-look complex images in the next section will be converted to multilook images to reduce the level of speckle noise. In Table 1, detailed information on the images is given. The incidence angle of the images at the center of the swath is 29° with a tolerance ≤0.9°. The acquisition time of the swath is approximately 7 s. Although HH polarization images are useful for monitoring urban features, the sensing time of the images is not ideal as traffic jams usually form during the evening in the city. In the next section, we explain how dense urban areas could reduce the quality of SAR images in terms of road monitoring.

## 3. Methodology

### 3.1. Pavement Monitoring

**Figure 2:**
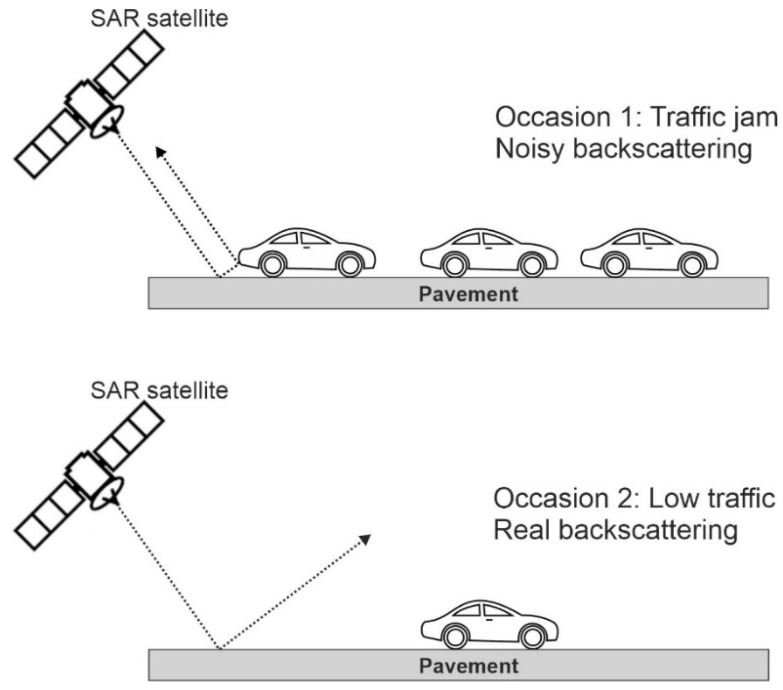
Schematic of SAR satellite geometry and backscattering coefficients of the pavement in the presence of a traffic jam (upper) and in the absence of a traffic jam (lower).

Image classification of high-resolution SAR data is useful to segregate interesting features. Buildings, ponds, and pavement are examples of common features in urban areas. The backscattering behavior of the features is different, but sometimes different features cannot be distinguished for several reasons. One reason is the shortcomings of the SAR systems: as they are side-looking systems, we might lose some features that are not in the illumination direction (e.g., shadows). Usually the backscattering of the buildings is strong, signal penetration into the buildings is not too high (unlike vegetation), and “double bounce” backscattering from the buildings is expected. In the case of natural events, the behavior of the buildings could also change. For example, if the buildings are not close to each other, a collapsed building’s backscattering coefficient will be reduced, but if the buildings are close to each other, the lowered backscattering of the collapsed building will be reflected from the side wall of the intact neighboring building [21]. The story is different for floods. The backscattering coefficient might increase in all SAR bands over vegetated areas, as volumetric backscattering behavior is affected by flooding. The results are heterogeneous for different case studies, so the developed methods must first be adapted to the local parameters [44]. Here, we aim to bear in mind the real backscattering behavior of buildings and pavement. The backscattering behavior of the buildings is rather strong in a dense city such Tabriz. Thus, the bright pixels in SAR images are representative of the buildings, or they are areas to be affected by the layover distortion. The layover problem occurs when a sharp feature such as a mountain interrupts the SAR illumination, especially when the foreslope (α) of a feature is larger than the incidence angle of the image (θ). We assume that the built-up area in Tabriz does not have a sudden slope. This assumption might help us to classify the buildings more easily. Since the main objective of this study is to extract the land subsidence rate of buildings and pavement, the SAR backscattering behavior of the pavement must also be characterized correctly. Usually, the quality of the pavement is related to the backscattering coefficient. If the pavement’s roughness is low, the pavement acts like a mirror and the transmitted energy from the sensor will be fully reflected in the opposite direction; like the behavior of water bodies, the backscattering coefficient will also be low. Thus, the road quality and moisture level are dependent on the backscattering coefficient. Thus, the backscattering is not solely representative of the pavement, but also of the double-bounce behavior of different vehicles on the pavement. Figure 2 shows two examples of SAR acquisition for pavement. In occasion 1 we see noisy backscattering of the pavement in the presence of a traffic jam and in occasion 2 we face real backscattering in the absence of a traffic jam. The acquisition time in Table 1 shows that the images over Tabriz were gathered during peak traffic.

**Figure 3:**
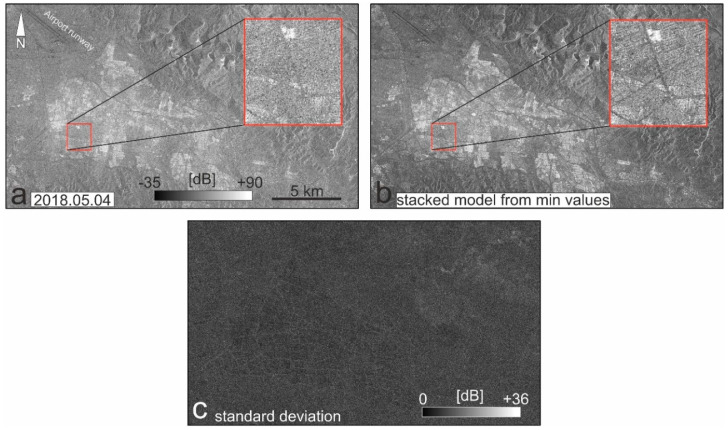
(**a**) Example of single look complex (SLC) SAR images over Tabriz (described in Table 1). Tabriz international airport’s runway in the upper left corner of the images is visible in dark pixels. As shown in the red square, the streets are not as dark as the runway, which means that either the pavement quality is lower than the runway or the number of vehicles on the streets is higher than the number of vehicles (e.g., plane) on the runway; (**b**) a minimum backscattering map of the pavement extracted from the stack of 15 images; (**c**) a standard deviation map of the study area from the stack of 15 images.

The image, taken on 4 May 2018, shows the buildings in brighter pixels, while the pavement is rarely visible (Figure 3a). The other sample images also show that the level of backscattering in the pavement is unexpectedly high. This implies that the temporal changes are not the main reason. On the other hand, the airport runway in the upper-left part of the city shows a considerably lower backscattering coefficient. The minimum and maximum backscattering coefficients for all 15 images are −35 dB and +90 dB, respectively. For the airport runway, the minimum and maximum backscattering coefficients of the images vary between +13 and +37 dB. The minimum and maximum values for the airport runway are recorded in images #5 and #3, respectively. Despite the low backscattering values of the runway, the behavior of the pavement is not similar, probably because of noisy backscattering due to the presence of more vehicles on the street.

**Figure 4:**
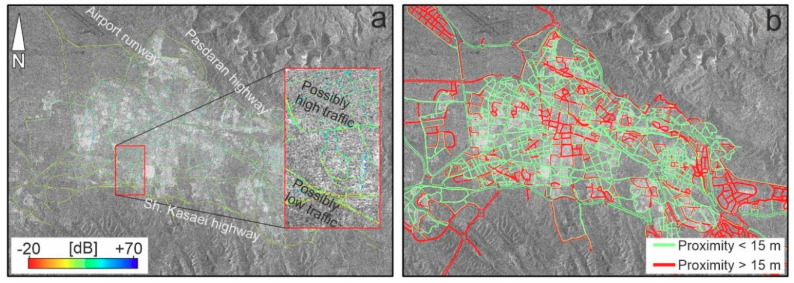
(**a**) Pavement map created by stacking the backscattering coefficient values. The red box magnifies a sample segment of the pavement network of the city; (**b**) proximity map of the roads.

**Figure A1:**
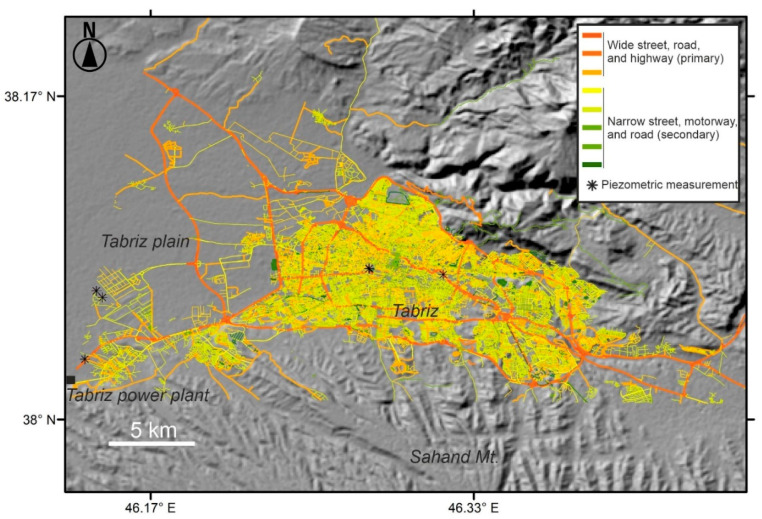
OpenStreetMap (OSM) of Tabriz and its surrounding area. Hot colors indicate wide streets and highways and cold colors indicate narrow streets or roads. Black squares and asterisks give the locations of power plant and piezometric sites, respectively.

We provided an image stacking solution in which all images over time are stacked. Image stacking is a way to combine several images to produce a single image for a certain purpose. The extent of the images (i.e., number of columns and rows) must be the same and all of the images that contributed to the stacking must be oriented or georeferenced with respect to a reference image or datum. If the images are not of the same resolution, a resampling process must be carried out before the image stacking. Considering all the obtained images, we have 15 different traffic occasions. Here, the idea behind the noise removal process is that the minimum backscattering coefficient of the images is more likely to result from the interaction of the transmitted signal and the pavement’s surface. Before the minimum-based stack method, we applied non-local means and Lee filters as two Speckle suppression approaches [45,46]. Figure 3b shows the denoised pavement overlaid on the mean image using image stacking. Comparing Figure 3a with Figure 3b shows that the selection of the lowest pixel value is helpful to visualize and classify the pavement. Figure 3c also shows that the standard deviation of the stacked images differs from 0 to 35 dB; most of the tolerance can be seen in the streets, layover-related distortions of sharp topographic feature, and shadows. Figure 4a is a pavement map of the study area created from the lowest backscattering coefficients for each representative pixel. Two main highways in the north and south of the city (Pasdaran and Shahid Kasaei) have rather low pixel values, so their asphalt quality could be higher. Since Pasdaran and Shahid Kasaei highways are wider international roads (~20 m), it is logical that they have higher quality. However, a low pixel value does not exclusively reflect the quality of the pavement. Probably, the remaining traffic noise in the city center and surrounding areas would have a negative impact on the pavement map. Thus, in Figure 4a, the hot pixel values probably represent a higher quality of pavement, lighter traffic, absence of tall buildings or all of these items. In contrast, the cold pixel values probably represent a lower quality of pavement, heavier traffic, presence of tall buildings or all of these items. In addition, we have carried out proximity analysis based on the extracted roads from the minimum-based stacking method and the primary and trunk roads of the OpenStreetMap (OSM) in GIS environment (Figure A1). The proximity analysis calculates Euclidean distance and proximity information between the extracted road map (input feature) and the OSM (reference feature). In order to extract a proximity map, we assign a search radius not larger than 15 m, which means that if the reference object is closer than 15 m, it will be reflected in the proximity map. Green lines in Figure 4b show that the main streets and road are recognized well and the standard deviation of the verified roads is 1.9 m. Must be noted that the red lines do not exist in the extracted map or they are not recognized well because their distances from the reference data were larger than 15 m.

**Figure 5:**
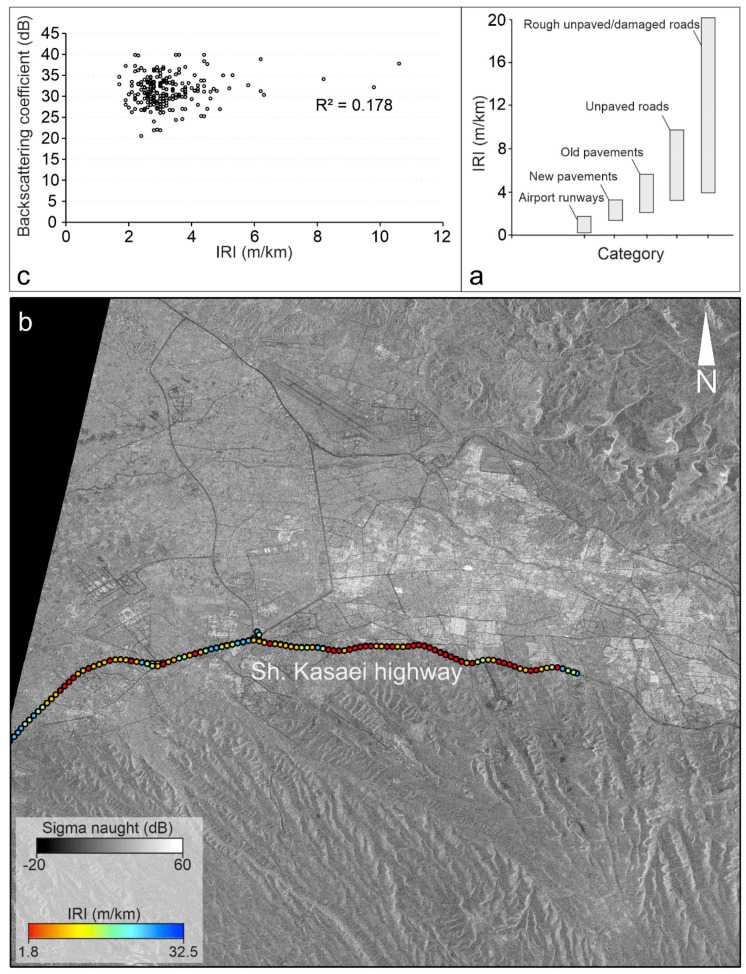
(**a**) International roughness index (IRI) and its relationship with different road categories [47]; (**b**) IRI of Shahid Kasaei highway measured by BumpRecorder application; (**c**) scatter plot for IRI measurements and backscattering coefficient.

We compare international roughness index (IRI) for the study area and descending SAR data. The IRI is one of the roughness indices that commonly has been used for description of the road quality by means of longitudinal profile of the roads. Figure 5a shows different road types and road qualities with respect to a specified IRI value range [47]. There are roughly three ways to measure IRI of the roads as follows: 1—IRI from precise one-wheel vehicles. In this technique a one-wheel vehicle that has not a suspension system like usual cars should be used to measure longitudinal profile. The sampling distance on the road should be no greater than 250 mm [48], 2—IRI from quarter car (QC) method. In this technique all types of usual vehicles or cars with four wheels can be used with a mobile application on the basis of the direct computations of the IRI after removal of effects of car suspension system. It must be noted that the accuracy of this method is acceptable for large scale projects, but its accuracy is not good enough for detailed projects. Typically the accelerometer sensors and a GPS device of a smart phone would be enough to measure inertial profile and distance, 3—IRI from indirect methods on the basis of correlation. In this technique the IRI can be computed from enough field data acquired from the method 1 or 2, and its correlation with other parameters.

Here, the precise IRI for the study area is not available. Thus, we measured the IRI in Kasaei highway using QC method and BumpRecorder application (Figure 5b,c). To avoid data redundancy, the IRI values are presented every 160 m. In order to do a fair comparison, we compare the IRI results with the last SAR (2019.04.21) image to keep the shortest temporal baseline between the IRI and the SAR backscattering coefficient. Must be noted that the IRI measurements with an average speed of 30 km/h and 100 Hz frequency were gathered on April 2020 and the SAR image values are adjusted by applying a mean 3 × 3 window size. Figure 5c shows the measured IRI and the scatter plot of the IRI values together with SAR backscattering coefficient values (2019.04.21). Intuitively, the correlation between IRI and backscattering coefficient is not surprising since the time difference between IRI and SAR data is about one year. Nevertheless, a very slight correlation can be observed between IRI and SAR backscattering coefficient values. As the IRI increases (road quality decreases), the backscattering coefficient increases. However, in order to achieve accurate results in the dense areas, the integration of ascending and descending SAR datasets can reduce negative impacts of the tall buildings near to the roads [21]. A previous study also showed that probably the relationship between these items is not linear exclusively and more sophisticated correlation analyses (e.g., exponential curve) show better results [47].

### 3.2. SBAS Time Series Analysis

Several time series approaches have been introduced for SAR data analysis [49,50,51]. Two main techniques of interferometric time series analyses are a permanent scatterer (PS) InSAR and a small baseline or SBAS InSAR [43,49]. Although SBAS is a technique, it can also be considered as an algorithm for SAR processing too. Previous studies revealed that SBAS was an effective technique to monitor land subsidence in Iran [30,31,32,33,34,35,52]. Here, we follow the SBAS InSAR method introduced by Berardino et al. to derive mean velocity displacement values. The analysis will be based on N+1 images with the same characteristics (e.g., orbit, track, mode) taken at specific times ($t_{0},\;\ldots .,t_{N}$). We assume that all the images are co-registered with respect to a “super master image” and that at least one image can contribute to the interferometric analysis. The super master image is an ideal master image that can satisfy both the temporal and normal baselines’ limitations. For example, the super master image should not be temporally far from the first or last images. Its relative spatial baseline to the other images should also not be too large. Each SBAS interferogram has at least two images. Thus, if we assume that N is an odd number, the number of differential interferograms (M) can be used to estimate the low-pass signal component as follows:

(1) $$\frac{N+1}{2}\leq M\leq N\left(\frac{N+1}{2}\right)$$

The radar coordinates of each pixel in the range and azimuth directions (x,r) of the produced unwrapped j-interferogram will contribute to the SAR images at times $t_{B}$ and $t_{A}$ as follows [50]:

(2) $$\varphi_{j}\left(x,\;r\right)=\varphi \left(t_{B},\;x,\;r\right)-\varphi \left(t_{A},\;x,\;r\right)\approx \frac{4\pi }{\lambda }\left[d^{LP}\left(t_{B},\;x,\;r\right)-d^{LP}\left(t_{A},\;x,\;r\right)\right]+\Delta \varphi_{j}^{atm}\left(t_{B},\;t_{A},\;x,\;r\right)+\Delta \varphi_{j}^{topo}\left(x,\;r\right)$$

where j is assumed to be an integer between 1 and M, $\varphi \left(t_{B},\;x,\;r\right)$; $\varphi \left(t_{A},\;x,\;r\right)$ are the associated multi-look phase components of the two images for the produced interferograms; $d^{LP}\left(t_{B},\;x,\;r\right)$ and $d^{LP}\left(t_{A},\;x,\;r\right)$ are the line-of-sight (LOS) deformations of the low-pass components accumulated from $t_{A}$ to $t_{B}$ with respect to the reference time ($t_{0}$); λ is the wavelength of the CSK satellite; $\Delta \varphi_{j}^{atm}\left(t_{B},\;t_{A},\;x,\;r\right)$ is the atmospheric phase associated between two acquisitions; and $\Delta \varphi_{j}^{topo}\left(x,\;r\right)$ is the topographic phase, mainly because of errors in the digital elevation model (DEM) and the Earth’s features, defined as follows:

(3) $$\Delta \varphi_{j}^{topo}\left(x,\;r\right)\approx \frac{4\pi }{\lambda }\frac{B_{⊥j}\Delta z\left(x,\;r\right)}{rsin\theta }$$

where $B_{⊥j}$ is the perpendicular baseline of the two images that contributed to the interferometric analysis, θ is the incidence angle of the images (~29° for CSK), and Δz(x, r) is a topographic artefact that can be reduced by the DEM. Note that the instrumental noise, such as from the overheating of the sensors, etc., is assumed to be zero. Thus, according to Equation (2), to achieve the pure deformation rate, the $\Delta \varphi_{j}^{atm}\left(t_{B},\;t_{A},\;x,\;r\right)$ and $\Delta \varphi_{j}^{topo}\left(x,\;r\right)$, components must be separated from the rest of the equation.

**Figure 6:**
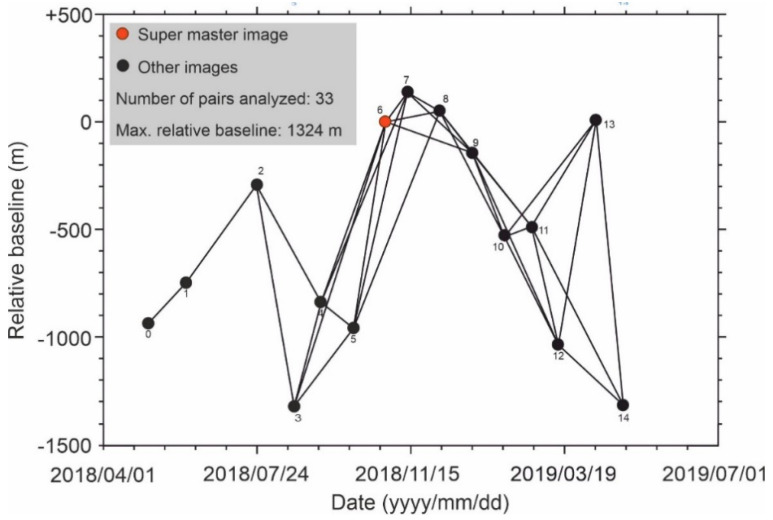
Temporal (up to 60 days) and normal baseline (up to 1324 m) graph of the CSK images used in the small baseline subset (SBAS) analysis from 4 May 2018 to 21 May 2019. The red circle is the super master image, which is used for co-registration processes; the black circles are other images that contributed to the SBAS analysis, and the black lines represent interferometric pairs of the images. The number of each circle is described as “label” in Table 1.

We used 1 arc-second (~30 m) Shuttle Radar Topography Mission (SRTM) DEM and 40 ground control points in motionless parts of the study area that pose high coherence values to reduce or remove the topographic and atmospheric effects. Among all pairs, the pair of images 4 and 6 had the lowest mean coherence (0.34) and the pair of images 7 and 8 had the highest mean coherence value (0.68). We have defined spatial and temporal constraints for the potential SBAS pairs to finish the InSAR processing in a timely manner and reduce the level of uncertainty. Here, the maximum temporal gap for a potential SBAS analysis is set to be 60 days and the normal baseline can be enlarged up to 50% of the critical baseline. Accordingly, we can maintain the integrity of the SBAS network without any separate network, as shown in Figure 6, and also avoid generating excessive SBAS pairs, which can increase the time of analysis. The minimum normal baseline belongs to the pair of images 7 and 8 (49 m) while the maximum normal baseline belongs to the pair of images 4 and 6 (1324 m).

### 3.3. InSAR Vertical Motion Estimation

**Figure 7:**
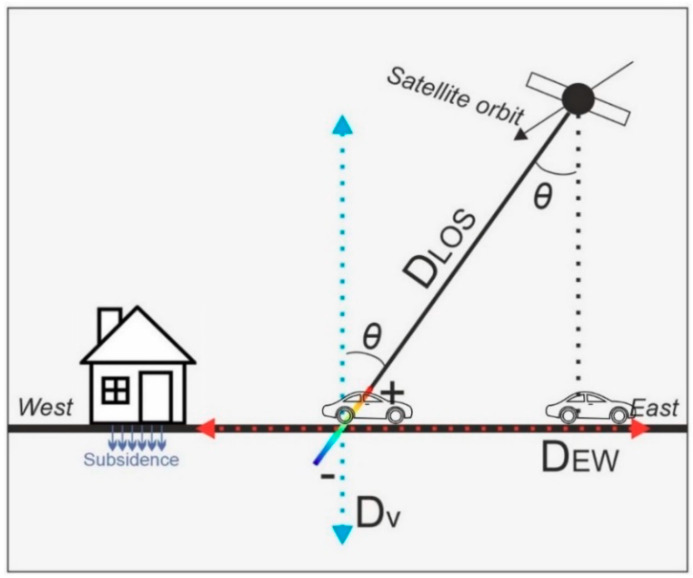
Interferometric SAR (InSAR) displacement components in the vicinity of land subsidence. θ is the line-of-sight (LOS) incidence angle of the satellite.

First, we note that, since the images are gathered only from descending orbits, the SBAS map provided would show one-dimensional displacement in the satellite-and-ground system. As explained in the previous subsections, the LOS direction is representative of the whole displacement, which is composed of three velocity vectors, D_EW_ and D_NS_ (horizontal vectors) and D_V_ (a vertical vector). The LOS displacement is a problematic component that cannot be compared with other geodetic observations such as leveling or GPS. Thus, for land subsidence characterization, it is reasonable to use the vertical component instead of line-of-sight displacements. On the other hand, the SBAS InSAR measurements are not very sensitive to north-south displacements because CSK satellites and other SAR satellites are polar orbiting satellites. Figure 7 displays InSAR displacement components in the vicinity of land subsidence.

We ignore D_NS_ vector (north–south movement) and convert the LOS movements or velocities to estimate D_V_ as follows:

(4) $$D_{v}=\frac{D_{LOS}+\;D_{EW}sin\theta cos\alpha }{cos\theta }$$

Here, $D_{LOS}$ is the LOS displacement, $D_{v}$ is the vertical displacement, $D_{EW}$ is the east–west displacement, α is the azimuthal angle of the LOS, and θ is the incidence angle of the satellite. We also assume that other tectonic or nontectonic local east-west displacements are trivial. Thus, the numerator of Equation (4) is $D_{LOS}$. This assumption is reasonable because we can expect that hydrostatic loads are mainly related to $D_{v}$. The relative vertical displacement is calculated with respect to a reference area (motionless). The reference area is chosen based on the following criteria: its distance from the land subsidence patterns or uplift areas; the relative coherence value of the produced interferograms, in which high coherence can be an ideal factor for reference area; and its position far from ground instabilities such as a landslide.

## 4. Results

### 4.1. InSAR Velocities

**Figure 8:**
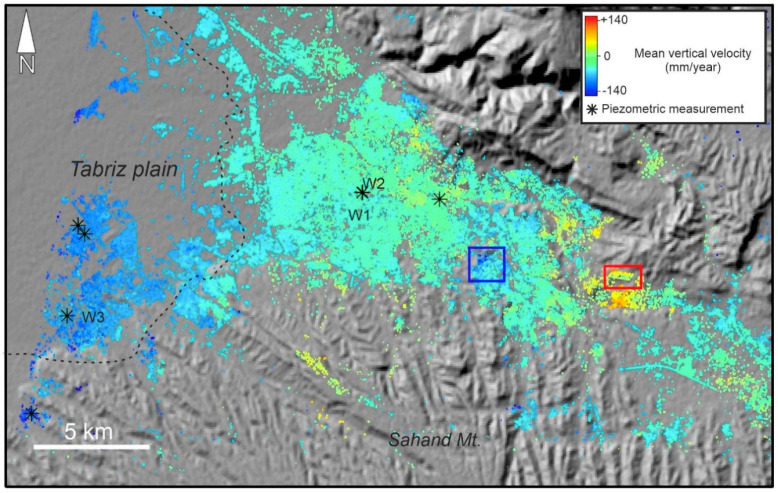
Mean CSK deformation velocity map of Tabriz in the vertical direction. The black asterisks are the piezometric sites. The dashed polygon represents Tabriz plain; the red and blue rectangles are enlarged in Figures 13 and 14, respectively. Detailed information on subsidence (blue rectangle) is given in Table 2.

**Figure 9:**
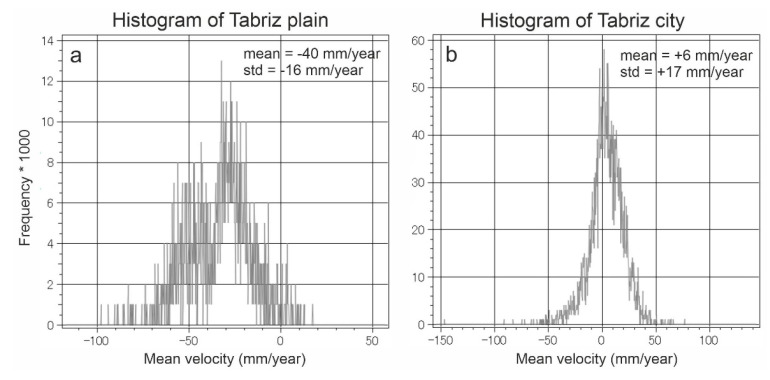
Statistical results of land deformation for (**a**) Tabriz plain and (**b**) Tabriz city.

**Figure 10:**
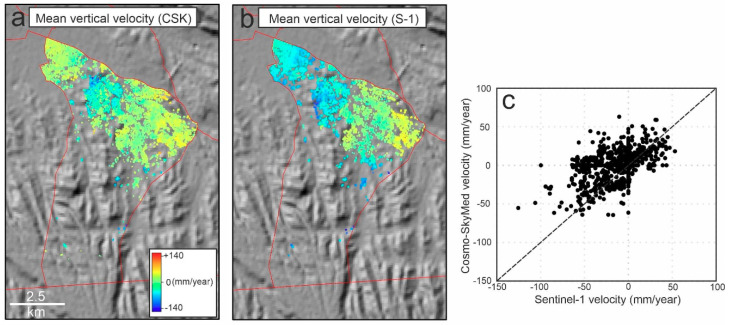
(**a**) CSK mean vertical velocity map of district two; (**b**) Sentinel-1 mean vertical velocity map of district two; (**c**) scatter plot of mean vertical velocities.

**Figure A2:**
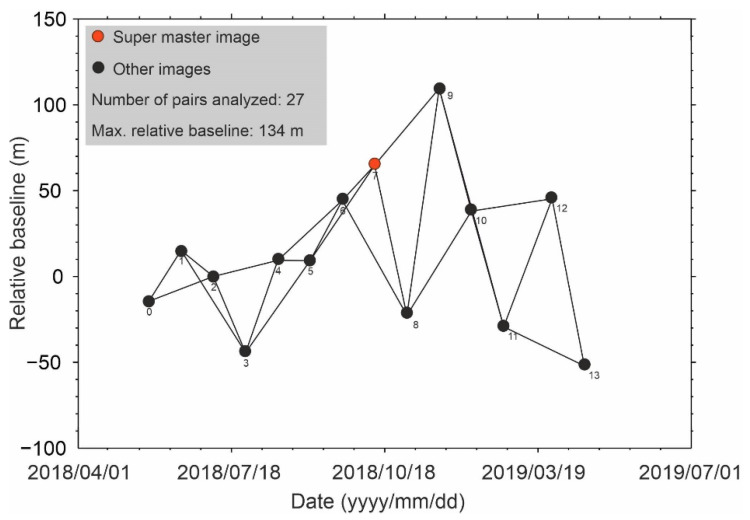
SBAS interferogram network of Sentinel-1 SAR dataset for co-polarization (VV) (from 23 May 2018 to 12 April 2019). The red circle is the super master image, which is used for co-registration processes; the black circles are other images that contributed to the SBAS analysis, and the black lines represent interferometric pairs of the images.

Figure 8 shows the SBAS InSAR mean velocity map of the study area from May 2018 to May 2019 using HR CSK data. Hot colors (positive values) and cold colors (negative values) indicate uplift and subsidence, respectively. In the western part, Tabriz plain (shown as a dashed polygon) experienced the maximum rate of subsidence (−117 mm/year). The observed deformation in the plain is probably the continuation of a progressive land subsidence between 2003 and 2010. The reason for the land subsidence was extensive water withdrawal for agricultural and industrial purposes [30,35,53]. It must be noted that in some parts of the plain (the dashed polygon), there are some uplifts reaching +56 mm/year. However, the corresponding histogram shows that the uplift is not significant (Figure 9a); the mean deformation rates and standard deviation on the plain were −40 mm/year and −16 mm/year, respectively. For the urban area, the minimum and maximum rates were −154 mm/year and +143 mm/year, respectively. Unlike the wide normal distribution of Tabriz plain, the normal distribution of the urban areas was sharp. As shown in Figure 9b, the peak of the normal distribution of the deformation values is close to zero (~+6 mm/year), which means that the city was almost stable and did not experience a uniform land subsidence, as was happening in the plain. However, there are some areas with subsidence (the blue rectangle in Figure 8). The subsidence area inside the city is located in district two (see Figure 1). In addition, the city has developed rapidly towards the east, where the probable uplift areas were observed. Since the GPS and leveling measurements are not available for the study area, we gathered field evidence related to the impacts of the subsidence on the buildings, and also analyzed 14 co-polarized (VV) descending (T79) images of Sentinel-1 from 23 May 2018 to 12 April 2019 using SBAS technique (Figure A2). The mean vertical velocity maps deduced from the HR CSK and Sentinel-1 datasets present similar patterns for district two (Figure 10). The correlation between the two maps is 0.77 and the HR CSK map resulted in higher mean velocities than the Sentinel-1 map.

**Figure 11:**
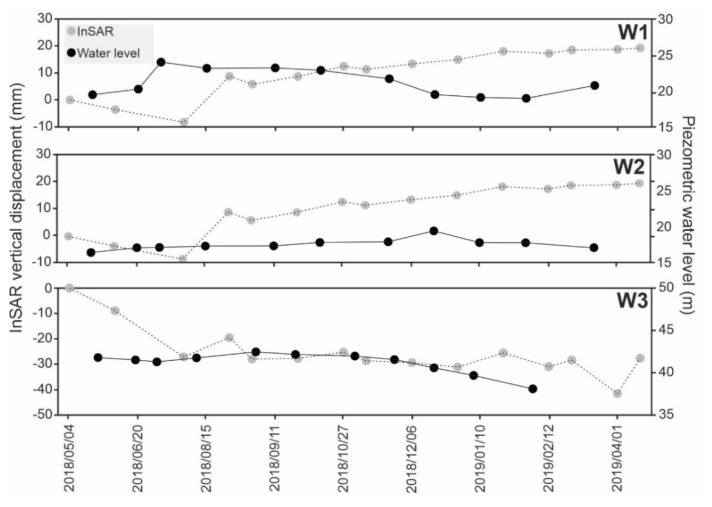
Comparison of water level fluctuations and InSAR vertical deformations in W1, W2 and W3. The locations of W1, W2 and W3 are shown in Figure 8.

The piezometric level of some wells in the study area has been gathered by RWO for more than one decade. Most of the piezometric records are from 2006 to 2018, which is not quite suitable for comparison to the CSK dataset (2018–2019). In addition, the majority of wells are located in the outskirts. Thus, only seven piezometric wells are selected, and only three of them are located inside the city. It must be noted that, since some of the selected wells were destroyed or filled in, we only use sites W1, W2 and W3 to compare their water level with the corresponding InSAR time series. As shown in Figure 11, the behavior of the underground water fluctuation versus InSAR deformation differed in W1, W2 and W3. Since W3 is located at the boundary of the city and the plain, the correlation between the underground water level and the vertical displacement was higher than in W1 and W2. In W3, the obvious underground water depletion led to land subsidence from 19 May 2018 to 13 March 2019 (Figure 11). In the W1 and W2 wells, the level of underground water fluctuations and the vertical displacements did not show a significant correlation, possibly because W1 and W2 are located in an urban region; they have a slight uplift, instead of a progressive land subsidence; and the underground water level and vertical land displacement are not exclusive indicators of the aquifer’s behavior. Although underground water fluctuations in W1 do not have a significant correlation with the vertical displacement, in W2, the uplift and underground water level has a good correlation between 15 August and 14 December 2018.

**Figure 12:**
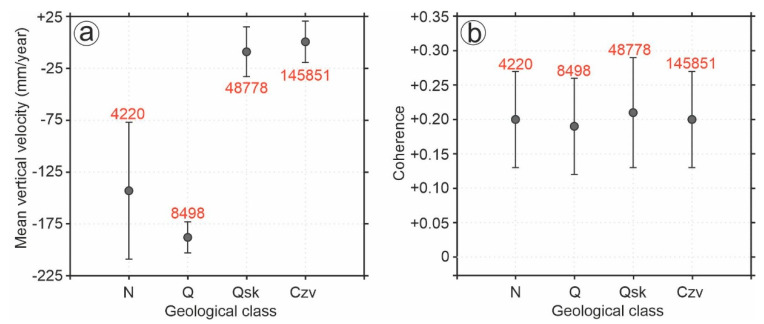
(**a**) Relationship between geological classes and deformation rate; (**b**) relationship between geological classes and interferometric coherence. The digits in red are the number of pixels in each class.

The study area is dominated by the Neogene, Cenozoic volcanic, and Quaternary classes. Here, the areas of land subsidence are in the Quaternary and Quaternary marsh classes. We examined the abundance of SBAS pixels and the corresponding mean velocity rate. The numbers of pixels in the Neogene, Quaternary, Quaternary marsh, and Cenozoic volcanic are 4220, 8498, 48,778, and 145,851, respectively. As shown in Figure 12a, the Quaternary class shows the highest land subsidence rate (−188 mm/year), and the corresponding standard deviation is 15 mm/year. This implies that the Quaternary class generally consists of fine-grained alluvial plane, so the most land subsidence is expected from this class. Since the subsidence is more commonly related to subsurface geology, further tests such as cone penetration testing (CPT) are necessary to identify the types of subsurface soil [54,55]. Figure 12b shows that the levels of coherence in all four dominant classes are close to each other. The coherence values for the Neogene, Quaternary, Quaternary marsh, and Cenozoic volcanic are 0.20, 0.19, 0.21 and 0.20, respectively. The standard deviation of the coherence values for the geological classes is almost the same (0.07).

### 4.2. Pavement, Buildings and Field Observations

**Figure 13:**
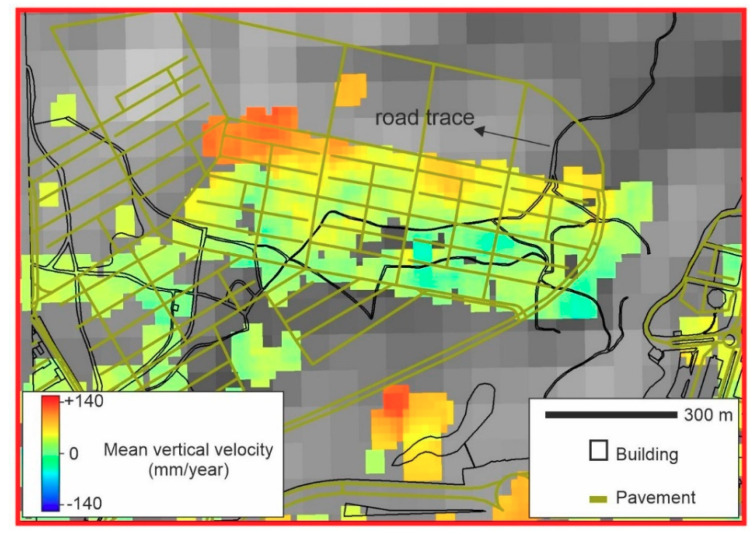
The uplift area in new towns located in the eastern part of Tabriz (Marzdaran).

**Figure 14:**
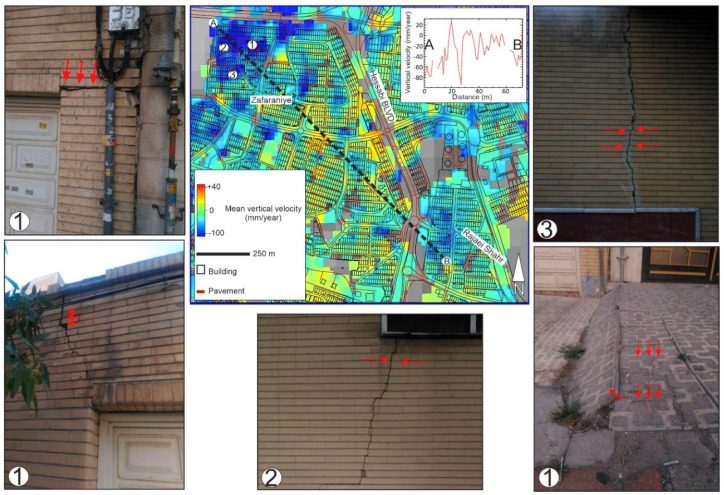
The subsidence area in Zafaraniye. Examples of damage such as cracks on the walls and deformed pavement show evidence of land subsidence.

Figure 13 shows red pixels in the eastern part of the city. The source of these positive values are unknown, if it is uplift, it is probably associated with urban growth and rapid construction projects or natural uplift due to the rising of the water level. If it is not uplift, the positive values are probably due to DEM artifacts. The mean deformation rate in the red rectangle in Figure 8 (the uplift area) is +53 mm/year, while the minimum and maximum deformation rates are +6 and +146 mm/year, respectively. According to recent landscape models, the built-up area will increase to 90% from 2005 to 2021 [56]. Figure 14 shows that the area northwest of Zafaraniye (the blue rectangle in Figure 8) is considerably affected by land subsidence. This could be partially due to underground water extraction, as there are many illegal deep wells here. Since the foundations of many buildings in this area are deformed, the traffic and structural load is also an important contributor to local land subsidence. Our field observations show that, because of the local subsidence, cracks have appeared on walls and there have been some major shifts of curbstones and tiles (Figure 14). It is difficult to quantify the contribution of the subsidence factors in this area because a small-scale geological map of the urban area is not available. The mean deformation rate in the blue rectangle is −20 mm/year. The minimum and maximum deformation rates within the blue rectangle are +10 mm/year and −150 mm/year, respectively. The pavement extracted from the image stacking method and auxiliary information contribute to the total length calculation. The total length of pavement affected by subsidence is 65 km. The subsidence area drawn in the blue rectangle also covers a dense built-up area of approximately 1.4 km^2^.

## 5. Discussion

As mentioned, the road roughness mapping using one-wheel or QC techniques is a time-consuming cost-intensive task. Thus, the comparison of the SAR backscattering values with IRI can be profitably used for developing new algorithms and road maintenance purposes in the future. However, in this study, the comparison does not prove exclusively that the road quality assessment is possible because the correlation between the backscattering coefficient and IRI values is not high and also we did not compare the results with other independent datasets. The backscattering coefficient could be one of the indicators of the road quality. For example in the U.S., secondary roads show a good correlation while the interstate roads show little-to-no correlation with X-band backscattering coefficient [47]. Thus, further works will reveal more aspects of the road mapping using SAR data.

In this study, our quota for X-band SAR dataset was limited up to 15 descending images. This is marginally enough for a time series SBAS analysis. We performed SBAS-InSAR analysis with an assumption that only vertical deformation is happening in the study area. Thus, the LOS results were converted to vertical velocity. As mentioned before, the positive vertical values are probably uplift areas, mainly associated with construction projects and land fillings. However, it could be related to the use of SRTM DEM from the year 2000 (before the buildings were constructed) for InSAR analysis of a period when buildings were already there. According to Crosetto et al., typically 15–20 images are enough for time series analysis; it is even possible to use short dataset for X-band data because of higher resolution and shorter wavelengths [43], but integration of ascending and descending datasets with further images is necessary for an ideal SBAS analysis in future studies to produce a 3D velocity map and reduce the level of uncertainties.

## 6. Conclusions

The main aims of the study were to assess pavement quality and the pavement area affected by land subsidence in urban areas using SAR images. To achieve these goals, we needed to examine the potential of SAR imagery for the segregation of pavements and buildings. The extraction of buildings is not difficult as they have double bounce backscattering coefficient. However, extraction of the pavement is challenging because traffic jams can seriously disrupt the backscattering signal. We tried applying a minimum-based image-stacking method along with proximity analysis to extract pavement from 15 HR SAR images. We conclude that the extraction of pavement is possible and additional data such as reference OSM data and polarimetric SAR data will be useful for precise quality assessments of the roads.

This study also showed that the progressive land subsidence observed from 2003 to 2010 is still continuing in the Tabriz plain. Without necessary action or water policies, the land subsidence could be harmful to critical infrastructure located in the plain, such as Tabriz power plant and Tabriz refinery. The immature local subsidence inside the urban area detected by CSK and Sentinel-1 datasets is also a potential threat to the buildings and pavement. Although cracks and minor damage to walls do not exist on all of the buildings, without continuous monitoring, the number of buildings or pavement damaged by urban subsidence will increase in the future. Thus, increasing the regular precise leveling operations and the number of GPS stations inside the city is recommended.

## Figures and Tables

**Table 1:** Detailed information on the CSK high resolution (HR) SAR images used in this study. “*” and “D” indicate super master image and descending orbits, respectively.

Label (#)	Date (YYYY/MM/DD)	Satellite ID	Incidence Angle (°)	Product ID	Polarization	Orbit	Time (Local)
0	2018/05/04	1	29	1075358	HH	D	18:20
1	2018/06/01	4	29	1075417	HH	D	18:20
2	2018/07/23	1	29	1075413	HH	D	18:20
3	2018/08/20	4	29	1075480	HH	D	18:20
4	2018/09/09	1	29	1075407	HH	D	18:20
5	2018/10/03	2	29	1075421	HH	D	18:20
6 *	2018/10/27	1	29	1075466	HH	D	18:20
7	2018/11/12	1	29	1075419	HH	D	18:20
8	2018/12/06	2	29	1075464	HH	D	18:20
9	2018/12/30	1	29	1075414	HH	D	18:20
10	2019/01/23	2	29	1075469	HH	D	18:20
11	2019/02/12	4	29	1075472	HH	D	18:20
12	2019/03/04	1	29	1075420	HH	D	18:20
13	2019/04/01	4	29	1075482	HH	D	18:20
14	2019/04/21	1	29	1075415	HH	D	18:20

**Table 2:** Detailed information on the pavement and buildings affected by subsidence for the areas in blue rectangles in Figure 8.

Target Area	Total Affected Pavement (km)	Total Affected Structure (km^2^)	Mean Deformation Rate (mm/year)	Maximum Deformation Rate (mm/year)	Minimum Deformation Rate (mm/year)
Subsidence Area (Blue)	65	1.4	−20	−150	+10
